# Could Inhibiting the DNA Damage Repair Checkpoint Rescue Immune-Checkpoint-Inhibitor-Resistant Endometrial Cancer?

**DOI:** 10.3390/jcm12083014

**Published:** 2023-04-20

**Authors:** Yinuo Li, Xiangyu Wang, Xin Hou, Xiangyi Ma

**Affiliations:** Department of Obstetrics and Gynecology, Tongji Hospital, Tongji Medical College, Huazhong University of Science and Technology, Wuhan 430030, China

**Keywords:** endometrial cancer, immunotherapy, DNA damage repair inhibitors, ATM-CHK2-P53, ATR-CHK1-WEE1, combination therapy

## Abstract

Endometrial cancer (EC) is increasingly undermining female health worldwide, with poor survival rates for advanced or recurrent/metastatic diseases. The application of immune checkpoint inhibitors (ICIs) has opened a window of opportunity for patients with first-line therapy failure. However, there is a subset of patients with endometrial cancer who remain insensitive to immunotherapy alone. Therefore, it is necessary to develop new therapeutic agents and further explore reliable combinational strategies to optimize the efficacy of immunotherapy. DNA damage repair (DDR) inhibitors as novel targeted drugs are able to generate genomic toxicity and induce cell death in solid tumors, including EC. Recently, growing evidence has demonstrated the DDR pathway modulates innate and adaptive immunity in tumors. In this review, we concentrate on the exploration of the intrinsic correlation between DDR pathways, especially the ATM-CHK2-P53 pathway and the ATR-CHK1-WEE1 pathway, and oncologic immune response, as well as the feasibility of adding DDR inhibitors to ICIs for the treatment of patients with advanced or recurrent/metastatic EC. We hope that this review will offer some beneficial references to the investigation of immunotherapy and provide a reasonable basis for “double-checkpoint inhibition” in EC.

## 1. Introduction

Endometrial cancer (EC) is one of the most common gynecologic malignancies, accounting for about 4.5% of female tumors, and its incidence is on the rise worldwide [1]. In developed countries, endometrial cancer is the leading malignant tumor of the reproductive tract in females, mainly due to the increased incidence of metabolic disorders. In the United States, endometrial cancer has become the most common female cancer, only inferior to breast cancer, with an estimated total of 291,560 cases in 2022 [2]. Due to the prevalence of high-sugar, high-fat diets and sedentary lifestyles, the incidence of endometrial cancer in China is increasing year by year. However, the survival rate for endometrial cancer has not improved over the past few decades, and the mortality rate has continued to increase, suggesting a lack of breakthroughs in treatment [3].

The majority of endometrial cancer cases are diagnosed at an early stage, and good clinical outcomes can be achieved with surgery alone (hysterectomy and bilateral salpingo-oophorectomy) or combined adjuvant therapy [4], with a 5-year survival rate of 74–91% [5]. However, about 15% of endometrial cancer cases are likely to recur, and advanced tumors show a higher degree of malignancy with a 5-year recurrence rate of up to 59% [6]. For patients with recurrent endometrial cancer, the prognosis is usually unfavorable. The 5-year survival rate is 55% in patients confined to pelvic recurrence but reduces to 17% in patients with distant metastases, and it is only about 10% in patients with multifocal recurrence [7], which is the leading cause of death in endometrial cancer and heavily affects the quality of life and clinical outcomes of patients. Moreover, once relapse occurs, traditional treatment, including surgery, radiotherapy, and chemotherapy, may fail to prolong the survival of patients with endometrial cancer in some cases. Therefore, it is urgent to explore more effective treatment methods to improve patients’ survival quality.

Immunotherapy, which uses immune cells in the patient’s body to attack tumor cells, is different from radiotherapy and chemotherapy and has the advantages of high efficacy, high specificity, and low adverse effects. Immunotherapy has emerged as a promising treatment for patients with malignant tumors for whom conventional treatments have not been effective. In endometrial cancer, a series of clinical trials have been conducted to evaluate the efficacy of immunotherapy in endometrial cancer, especially in advanced or recurrent/metastatic tumors. However, it was found that only a fraction of endometrial cancers responded to immunotherapy alone, and most patients did not achieve satisfactory treatment results. Targeted therapies that target protein molecules inside tumor cells can specifically select oncogenic sites and exert specific antitumor effects without affecting surrounding normal tissue cells. Among the targeted therapies, targeted inhibitors of DNA damage repair (DDR) are widely available and most promising. DDR inhibitors are expected to improve the tumor microenvironment in patients with endometrial cancer and reawaken the sensitivity of immune-checkpoint-inhibitor-resistant endometrial cancer to immunotherapy. Therefore, on the one hand, this review summarizes the current status of research on immunotherapy in endometrial cancer. On the other hand, it summarizes the potential applications of DDR inhibitors in oncology, especially in endometrial cancer. We focus on the mechanism of the interaction between the DDR pathway and tumor immunity and highlight the prospective application of several DDR inhibitors in endometrial cancer, especially in combination with immunotherapy for immune-checkpoint-inhibitor-resistant endometrial cancer, to improve the quality of patient survival and enhance the long-term survival of patients.

## 2. Immunotherapy in Endometrial Cancer: Progress and Problems

Endometrial cancer is traditionally divided into two types: type I (estrogen-dependent) and type II (estrogen-independent) [8]. With the development of multiomics technology, The Cancer Genome Atlas (TCGA) proposed the molecular classification of endometrial cancer in 2013 [9], which contains four main subtypes: (1) POLE-hypermutation; (2) MSI-H/dMMR; (3) copy number low (CNL)/NSMP; and (4) copy number high (CNH)/p53abn, closely related to the prognosis of patients, among which, POLE-hypermutation has the best prognosis, followed by MSI and CNL, and in contrast, CNH endometrial cancer accompanied by frequent p53 mutations has the worst prognosis. Molecular classification marks the era of precision treatment for endometrial cancer. Immunotherapy for endometrial cancer with specific molecular characteristics has been verified by gradually accumulated clinical evidence.

### 2.1. Clinical Efficacy of Immunotherapy in Endometrial Cancer

The novel molecular classification of endometrial cancer not only provides important prognostic information but also generates biologically defined subgroups where the tumors may respond differently to specific drugs, such as immune checkpoint inhibitors (ICIs). For example, POLE-mutation and MSI-H/dMMR endometrial carcinomas are associated with high tumor mutation burden (TMB) and significantly increase immune cell infiltration, which are excellent indications for immune checkpoint blockade strategies, particularly anti-PD-1/PD-L1 therapy [10,11]. In 2017, the FDA approved pembrolizumab (anti-PD-1 monoclonal antibody) for the treatment of patients with MSI-H/dMMR advanced solid tumors (including endometrial cancer) who had experienced disease progression before immunotherapy but had no reliable alternative treatment regimens, and 78% of patients reached progression-free survival (PFS) at 6 months after pembrolizumab treatment, regardless of tumor origins, which had led to a rapid expansion of clinical research on ICIs in endometrial cancer [12]. The KEYNOTE-158 study recruited patients with advanced MSI-H/dMMR solid tumors who failed with previous treatment, including 49 patients with endometrial cancer, and they were given an intravenous injection of 200 mg of pembrolizumab every 3 weeks. As a result, pembrolizumab monotherapy showed strong antitumor activity in MSI-H/dMMR advanced endometrial carcinoma with an objective response rate (ORR) of 48% and PFS of 13.1 months [13]. Dostarlimab is also an anti-PD-1 antibody. The GARNET study began to enroll patients with dMMR endometrial cancer in May 2017; 104 patients in total were eligible, and a final total of 71 patients with evaluable efficacy were included in the analysis. It was estimated that 96.4% of patients showed a maintenance of remission at 6 months and 76.8% at 12 months, indicating that dostarlimab has durable antitumor activity in MSI endometrial carcinoma and is a promising treatment with acceptable drug safety for MSI endometrial cancer that does not respond to other treatments [14]. Nivolumab is a PD-1 antibody for the treatment of unresectable or metastatic melanoma or non-small cell lung cancer (NSCLC) [15,16]. Azad et al. [17] evaluated the clinical activity of nivolumab in 42 patients with MSI tumors (including endometrial cancer), and the median overall survival (OS) of the patients reached 17.3 months, and the ORR was 36%. In addition to this, two clinical trials investigating nivolumab in endometrial cancer are ongoing (NCT02982486; NCT04106414). These data suggest that research related to nivolumab has been gradually increasing in endometrial cancer. However, as the studies progressed, some researchers found that T-cell immunoglobulin domain and mucin domain-3 (TIM-3) expression was upregulated in samples that developed resistance to PD-1/PD-L1 inhibitors. Hollebecque et al. [18] evaluated the efficacy of LY3300054 (anti-PD-L1 antibody) alone or in combination with LY3321367 (anti-TIM-3 antibody) in 82 patients with MSI-H/dMMR advanced solid tumors, including 14 patients with endometrial cancer. The results showed that the ORR of LY3300054 monotherapy was 32.5%, but the combination therapy reached 45%, indicating that anti-TIM-3 immunotherapy could partially reverse immune tolerance caused by anti-PD-1/PD-L1 alone, which should be focused on in subsequent studies. 

### 2.2. Limitations of Current Immunotherapy in Endometrial Cancer

Though ICIs have shown reliable antitumor activity in endometrial cancer, there are still some challenges. POLE- mutation endometrial cancer with a good prognosis demonstrates great sensitivity to immunotherapy with strong efficacy generated by monotherapy, but it only accounts for a small percentage of endometrial cancer and rarely recurs. Compared to microsatellite stable (MSS) endometrial cancer, MSI EC has a higher response rate to immunotherapy. However, it only accounts for 16% of recurrent endometrial cancer [19]. Moreover, due to the different mechanisms leading to microsatellite instability, MSI endometrial cancers are divided into sporadic MLH1 hypermethylation and Lynch-syndrome-associated EC, and these two groups respond differently to immunotherapy. In 2017, a review on clinical cancer research indicated that PD-L1^+^ cells were enriched in the patients with sporadic MLH1 hypermethylation. However, Lynch syndrome was characterized by increased CD8^+^ T cells but decreased PD-L1^+^ macrophages, suggesting that patients with Lynch syndrome EC was less responsive to single-agent anti-PD-L1 or anti-PD-1 therapies [20]. Therefore, for patients with Lynch syndrome, immunotherapy in combination with other agents may be necessary. Subsequently, an article in 2020 noted that there were significantly more PD-1^+^, CD8^+^, and CD45RO^+^ immune cells in Lynch syndrome EC than sporadic MLH1 hypermethylation tumors, and data from this article supported the possibility that Lynch syndrome might have a sustained antitumor immune response that is more sensitive to immune checkpoint blockade [21]. However, we should approach this issue in a comprehensive and integrated manner, and Zhao et al. [22] also dialectically explained the immune infiltration in sporadic MLH1 hypermethylation and Lynch-syndrome-associated EC. Therefore, ongoing immunotherapy trials must evaluate the outcomes of sporadic and Lynch syndrome endometrial cancers separately and explore more effective combination treatment strategies for patients in whom immune checkpoint inhibitors alone fail to exert antitumor effects. CNL/NSMP and CNH/P53abn tumors (MSS EC) with a poor prognosis make up a large proportion of endometrial cancer, which are at high risk of recurrence or metastasis after initial treatment. Immunotherapy as monotherapy has not been shown to have confirmed therapeutic benefits in patients with CNL/NSMP or CNH/P53abn tumors. Fortunately, the combination of pembrolizumab and lenvatinib showed an improvement in PFS and OS in this group of patients. Molecular signatures are critical in selecting immunotherapy in endometrial cancer; however, ICIs have limited efficacy in molecularly unselected patients with advanced or relapsed/metastatic endometrial cancer. In addition, the heterogeneity of the tumor can affect the response to treatment, and our knowledge of the oncologic biology is often limited to the analysis of a little tumor piece that cannot be representative of the whole lesion.

In addition to patients with MSI endometrial cancer, there are also studies exploring the application of immunotherapy in MSS endometrial cancer, but the effects vary. In the KEYNOTE-146 study, pembrolizumab in combination with lenvatinib improved survival outcomes in patients with MSI/dMMR or MSS/pMMR advanced endometrial cancer [23], which was determined by the FDA to be a groundbreaking therapy for advanced or metastatic non-MSI-H/dMMR endometrial cancer [24]. However, in the GARNET study, patients with recurrent or advanced endometrial cancer whose disease progressed during or after platinum-containing chemotherapy were treated with dostarlimab monotherapy, which produced an ORR of 43.5% in the MSI cohort, but only 14.1% in MSS endometrial cancer. Though it showed clinical significance in both cohorts irrespective of the MSI status, the efficacy of dostarlimab was unsatisfactory in the MSS cohort [25]. Similarly, a non-randomized, phase II clinical trial evaluated the clinical efficacy of durvalumab (anti-PD-L1 antibody) in MSI and MSS advanced or recurrent endometrial cancer, which included 35 MSI patients and 36 MSS patients, respectively, with an ORR of 47%, median PFS of 8.3 months, and OS rate of 71% at 12 months in the MSI cohort, compared with an ORR of only 3%, median PFS of 1.8 months, and 12-month OS of only 51% in the MSS cohort, which was significantly lower than the MSI cohort [26]. A clinical trial investigated the efficacy of PARP inhibitors talazoparib in combination with the PD-L1 inhibitor avelumab in 35 patients with recurrent MSS endometrial cancer, with a relatively low ORR (11.4%) and 6-month PFS rate (22.9%) [27]. These data suggest that there are still some obstacles to the widespread application of immunotherapy in endometrial cancer. Additionally, the current clinical studies have not shown satisfactory antitumor efficacy in patients with advanced or recurrent/metastatic disease who require more personalized treatment. Therefore, more research is warranted to explore the use of ICIs in patients with advanced and recurrent/ metastatic endometrial cancer beyond the POLE and MSI subgroups and develop novel effective precision treatment regimens as monotherapy or combined with immunotherapy in endometrial cancer.

The above content summarizes the progression of immunotherapy in endometrial cancer and objectively evaluates its effectiveness and limitations. The clinical studies of immunotherapy in endometrial cancer are listed in Table 1, which help us to understand the research trends of immunotherapy in endometrial cancer more systematically.

### 2.3. Prognostic Biomarker in Endometrial Cancer

Mismatch repair status can predict the clinical benefit of immunotherapy [32]. As the research progresses, the discovery of other markers is critical for predicting the response of tumors to immunotherapy. PD-L1, expressed in tumor cells, can initiate the programmed death of T cells by binding to PD-1 so that tumor cells achieve immune escape. There is growing evidence that PD-L1 expression is associated with the effectiveness of immunotherapy and can be used as a biomarker for predicting efficacy [33]. In melanoma, the ORR to anti-PD-1 therapy in patients with PD-L1-overexpressed tumors was 44–51%, compared with 6–17% in patients with PD-L1-negative tumors [34]. Likewise, the response rate of immunotherapy in patients with PD-L1-overexpressed NSCLC ranged from 67% to 100%, compared to a significantly lower response rate of 0–15% in PD-L1-negative NSCLC [35]. 

KEYNOTE-028, a multicohort phase Ib clinical study, enrolled 24 patients with PD-L1-positive advanced endometrial cancer with one patient distinguished by POLE mutation, one MSI, and the other patients undefined, all receiving pembrolizumab treatment. Pembrolizumab showed good safety and durable antitumor activity in patients with advanced PD-L1-positive endometrial cancer [28]. This study suggests that PD-L1 is a reliable biomarker for endometrial cancer immunotherapy, which is complementary to molecular classification and is expected to promote the application of immunotherapy in endometrial cancer. However, it is worth noting that PD-L1 as a predictive marker has some problems to be solved. For example, the FDA has approved the detection of PD-L1 expression by immunohistochemistry as an accompanying diagnosis to guide the treatment of gastric cancer, urothelial carcinoma and NSCLC [36,37,38], but the standard detecting methods need to be unified. Moreover, PD-L1 is widely expressed in tumor cells, and there is no gold standard to define its cut-off value related to the efficacy of immunotherapy, which is also a problem to be solved in the future.

In onco-genomics, TMB, defined as the total number of mutations in each coding region, is another promising biomarker [39]. Previous studies had comprehensively evaluated the relationship between TMB and ORR of immunotherapy in 27 tumor types and found that there was a positive correlation between TMB and ORR, and half of the difference in ORR among different tumor types might be related to TMB [40]. Therefore, we speculate that the response to immunotherapy is enhanced with increasing TMB. In 2014, TMB was proposed as a predictor for immunotherapy in melanoma [41], which had subsequently been extensively investigated in a variety of tumors. These data suggest that immunotherapy is appropriate in TMB-H endometrial cancers. Especially, DNA replication fidelity disruption and DNA repair defects result in high TMB in POLE mutations and MSI endometrial cancer, respectively [9]. However, one study reported that only 16% of patients with high TMB were defined as the MSI subtype, suggesting that there were also cases suitable for immunotherapy apart from MSI endometrial cancer, which would require further investigation in the future to promote immunotherapy towards precision and extensiveness [42].

Tumor-infiltrating lymphocytes (TILs) are a heterogeneous collection of lymphocytes with specific antitumor immune responsiveness. Prior studies had demonstrated TILs were associated with better tumor prognoses. In 2017, Sudo et al. [43] found that TIL status was a potent predictor of patients’ prognosis in esophageal cancer treated with surgery alone or combined with adjuvant chemoradiotherapy. TILs played a positively critical role in mediating chemotherapy response and improved clinical outcomes across all subtypes of breast cancer [44]. In endometrial cancer, Jong et al. [45] evaluated the prognostic value of TIL cells such as CD8^+^ T cells and FOXP3^+^ T cells. The results showed that TILs were an independent predictor of endometrial cancer and were associated with prolonged disease-free survival and OS. Immunotherapy restores the activation of T cells, which are involved in tumor cell recognition. TILs exert antitumor effects after initiation, so TILs can be used as an important predictive marker in immunotherapy. In recent years, TIL therapy has become an emerging immunotherapy that isolates T cells in tumor tissue in an orderly manner, expands them in vitro, and infuses them back into patients, thereby enhancing the immune response. TIL therapy could kill tumor cells without damaging the patient’s normal cells, in which case, TIL is a powerful marker for predicting efficacy.

With the advancement of immunotherapy, prognostic markers have also been widely studied. Besides the several markers mentioned above, other markers are also being discovered, which are not a single substance but interact with each other in a complex way. The combination of different markers will be necessary to predict treatment efficacy, and by being combined with molecular classification, they can be used to identify the most appropriate patients for immunotherapy. 

### 2.4. New Approaches to Improve the Efficacy of Immunotherapy and Enhance Antitumor Immunity

Despite impressive breakthroughs, there may be still a large proportion of endometrial cancer patients who are insensitive to immunotherapy alone, such as Lynch syndrome and NSMP or P53abn endometrial cancer. Patients with Lynch syndrome are usually complicated by colorectal and ovarian cancers, who have a greatly increased mortality rate. Most NSMP or P53abn endometrial cancers are advanced or recurrent/metastatic tumors and require more individualized treatment to improve the quality of survival and ameliorate long-term survival. Therefore, combinatorial therapy strategies are being developed to achieve a higher clinical benefit, including ICIs plus radiotherapy, chemotherapy, antiangiogenic agents, PARP inhibitors, and other targeted drugs.

The combination of immunotherapy and antiangiogenic drugs is a promising treatment strategy for endometrial cancer. As mentioned earlier, pembrolizumab in combination with the antiangiogenic agent lenvatinib improved survival outcomes in patients with advanced endometrial cancer and had been approved by the FDA for the treatment of patients with advanced or metastatic non-dMMR/MSI-H endometrial cancer [24]. In 2022, Wei et al. [46] exploratorily applied sintilimab and anlotinib in 23 patients with recurrent or advanced endometrial cancer, with an ORR of 73.9% and a disease control rate of 91.3%. The latest study found that DDR defects affect the antitumor immune response [47,48], highlighting that targeting the DDR could be a promising therapeutic strategy to promote the efficacy of immunotherapy in tumor. PARP inhibitors had shown synergistic lethality effects in tumors with DDR defects [49,50], which led to the expansion of the DDR pathway inhibitors [51]. In recent years, PARP inhibitors combined with immunotherapy had also been widely studied in endometrial cancer, but the effect was not satisfactory. As mentioned earlier, the combination of PARP inhibitors talazoparib and avelumab in recurrent MSS endometrial cancer produced a relatively low ORR and PFS at 6 months of only 22.9% [27]. Post et al. [52] found that durvalumab plus olaparib in patients with advanced endometrial cancer did not reach the scheduled 6-month PFS of 50%, despite good tolerance.

Besides PARP inhibitors, there are other DDR inhibitors considered emerging targeted agents, which are expected to be effective in immune-checkpoint-inhibitor-resistant tumors and synergy with immunotherapy to improve the survival outcomes of endometrial cancer with poor prognosis. This review will focus on the mechanism of the interaction between the DDR pathway and tumor immunity, and the application potential of DDR inhibitors combined with ICIs in the treatment of endometrial cancer, to provide new approaches for establishing optimal treatment strategies for immune-checkpoint-inhibitor-resistant endometrial cancer. 

## 3. DNA Damage Response Checkpoint in Endometrial Cancer

### 3.1. DNA Damage Response Checkpoint Inhibitors

Tumor cells will produce a large amount of damaged DNA during the replication process, which can activate a series of DNA repair mechanisms, mediated by DDR pathways, to maintain the genomic stability of tumor cells. Classical chemo/radiotherapy exerts antitumor effects by inducing DNA damage, and the activation of the DDR pathway is considered to be an important cause of drug resistance, so targeting DDR is an emerging antitumor treatment. The ATM/CHK2/P53 pathway and the ATR/CHK1/WEE1 pathway are the two main pathways that mediate DDR signaling transduction. Targeting key checkpoint kinases in the DDR pathway, such as ATM, ATR, CHK1/2, and WEE1, can hinder the process of DNA repair and promote cell killing in tumors. The combination of DDR checkpoint inhibitors and immunotherapy has been investigated in preclinical and/or clinical studies in a variety of solid tumors, which is expected to expand the application of immunotherapy and create a novel treatment option for refractory endometrial cancer.

#### 3.1.1. ATM/CHK2/P53 Pathway Inhibitors

The ATM/CHK2/P53 pathway mainly senses DNA double-strand breaks (DSBs), regulates G1/S cell cycle checkpoints by inhibiting the activity of CDK, and is involved in the repair process of DSBs [49]. Thus, the inhibition of ATM or CHK2 kinase inactivates the G1/S checkpoint and hinders DSBs repair, inducing apoptosis. 

ATM inhibitors include AZD1390, M3541, M4076, AZD0156, KU60019, and KU55933, etc., with potential chemoradiotherapy sensitization and antitumor activity [53]. AZD1390 is an oral ATM inhibitor. Compared with radiotherapy alone, the combination of AZD1390 and radiotherapy significantly promoted tumor regression and animal survival in homologous patient-derived glioma, as well as orthotopic lung–brain metastatic models [54]. M3541 had high selectivity and showed more significant antitumor activity in ATM wild-type tumor cell lines, suggesting that its inhibitory effect depended on the normal function of ATM. In an NSCLC cell line (A549), M3541 inhibited ATM and its downstream CHK2 and P53 activities in a concentration-dependent manner, resulting in the substantial accumulation of DSBs and sensitizing cancer cells to radiation. In addition, M3541 strongly enhanced the genotoxicity of radiation in four tumor xenograft mouse models (FaDu-head and neck, NCI-H1975-lung, Capan-1-colorectal, and NCI-H460-lung). Additionally, 3/4 of the models displayed complete and durable tumor regression, and no tumor regrowth was observed during treatment [55]. A phase I dose-escalation trial evaluated the safety and antitumor activity of M3541 in combination with palliative radiotherapy in patients with advanced solid tumors, finding that 20% of patients achieved partial or complete remission, without ≥ grade 4 events or treatment termination [56]. M4076 is a similar but more potent ATM inhibitor to M3541. In the 6-week FaDu mouse model, the powerful activity of M4076 combined with radiation completely regressed a large proportion of animal tumors during the study, suggesting that it could be the drug of choice for clinical studies [55]. AZD0156 is also an ATM inhibitor that enhances the sensitivity of melanoma cells to radiotherapy, without increasing damage to normal fibroblasts except for inducing cell death [57]. In a colorectal cancer (CRC) PDX model, AZD0156 suppressed irinotecan-induced DDR, and the combination of the two agents notably enhanced the inhibition of tumor growth [58]. Moreover, AZD0156 could reverse the chemotherapy resistance generated by ATM activation in neuroblastoma cells with telomere elongation [59]. Another ATM inhibitor, KU60019, was able to effectively decrease the expression of p-ATM and p-CHK2, which are markers of ATM inactivation. In endometrial cancer cell lines (HEC-1-B and HEC-6), KU60019 + DXR or CDDP, other than DXR or CDDP alone, was more likely to enhance cell killing. Subsequently, KU60019 was also observed to exert radiotherapeutic sensitization by inhibiting the radiation-induced activation of the ATM/CHK1 pathway [60]. More data indicated that the ATM inhibitor KU55933 remarkably increased the sensitivity of ovarian, cervical, and endometrial cancer cell line models to ionizing radiation therapy, without regard to P53 status, but failed to improve the tumor-killing effect of platinum-based drugs [61]. The data above represent the progression of ATM inhibitors in tumor treatment. ATM inhibitors have good antitumor activity and clinical application potential of radio-/chemo-sensitization, and they also exhibit preliminary activity in endometrial cancer, which needs to be further verified in clinical research.

The CHK2 kinase is located downstream of the ATM kinase and can be stimulated by a DNA damage signal, leading to cell cycle arrest or cell death. CHK2 inhibitors that can block CHK2 activation and the DNA damage response have been evaluated in basic research and clinical trials in a variety of solid tumors. Jobson et al. [62] synthesized PV1019 (NSC 744039) based on 4,4’-diacetyldiphenylurea-bis(guanosylhydrazone), which prevented the autophosphorylation of CHK2 kinase (defining the activated status of CHK2) by competitively binding to ATP in vitro. The study found that PV1019 suppressed cell proliferation synergistic with topotecan, camptothecin, and radiation in tumor cells, and protected normal mouse thymus cells from radiotoxicity. AZD7762 is a CHK1/2 inhibitor that effectively reduced the proliferation and sphere-forming capacity of three breast cancer cell lines (4T1.2, MDA-MB-231, and MCF-7), which showed dose-dependent activity of tumor suppression and might be linked to enhanced apoptosis and autophagy in the 4T1.2 cell line [63]. However, a phase I dose-escalation study reported that AZD7762 + gemcitabine yielded limited efficacy in patients with metastatic or unresectable advanced solid tumors, and the risk of adverse cardiotoxicity outweighed the treatment benefit, resulting in research discontinuation [64], which suggests that more clinical trials are needed to determine whether AZD7762 can be applied for the treatment of human tumors. BML-277 (a CHK2 inhibitor) was reported to inhibit the growth of laryngeal squamous cell cancer cells [65]. Likewise, Hseih et al. [66] found that BML-277 successfully inhibited the growth of oxaliplatin-resistant CRC cells in vitro and in vivo tumor models. Moreover, other CHK2 inhibitors are being developed simultaneously. Galal et al. [67,68] synthesized a variety of pyrimidine–benzimidazole conjugates for the CHK2 kinase, detected their effects on CHK2 activity via a checkpoint kinase assay, and preliminarily evaluated the antitumor capacity of CHK2 inhibitor monotherapy or combined with different genotoxic drugs in vitro. There are relatively few studies of CHK2 inhibitors in gynecologic tumors. Park et al. [69] are evaluating the safety and efficacy of PHI-101, an oral small molecule CHK2 inhibitor, in platinum-resistant recurrent ovarian cancer, but the findings are awaited. The existing data illustrate that CHK2 inhibitors have potential antitumor ability and can produce synergistic cytotoxic effects in combination with other drugs, but as one of the research directions in the future, research into their role endometrial cancer needs to be carried out. Especially for recurrent tumors, CHK2 inhibitors may be an important targeted drug to improve the survival of patients, and their combination with immunotherapy is expected to increase antitumor activity with minimal drug toxicity.

Many tumors harboring P53 deficiencies predominantly rely on G2/M cell cycle checkpoints to repair damaged DNA. The inhibition of the ATM/CHK2/P53 pathway alone may be not sufficient to eliminate tumor cells, so drugs targeting the ATR/CHK1/WEE1 pathway have been simultaneously developed to serve as an effective treatment for immune-checkpoint-inhibitor-resistant endometrial cancer. 

#### 3.1.2. ATR/CHK1/WEE1 Pathway Inhibitors

The ATR/CHK1/WEE1 pathway mainly senses DNA single-strand breaks and replication stress, regulates cell cycle checkpoints in the S phase and G2/M phase by CDK1 and CDK2, and participates in DDR through nucleotide excision repair, homologous recombination repair, and replication fork stabilization [49]. At the same time, tumors with ATM or P53 deficiency are highly dependent on the ATR/CHK1/WEE1 pathway to repair DNA damage to maintain genomic stability. Thus, the inhibition of ATR, CHK1, or WEE1 will affect cell cycle progression, inducing mitotic catastrophe and even cell death.

ATR is an important member of the PIKK family and plays a central role in the DDR. Several ATR inhibitors have been studied in preclinical or phase I/II clinical trials. Berzosertib (or M6620/VX-970/VE-822) is a potent small-molecule inhibitor of ATR. Berzosertib in combination with cisplatin or gemcitabine (±cisplatin) showed preliminary antitumor activity in resistant or refractory advanced solid tumors [70,71]. A phase Ib clinical trial reported an ORR of 23.4% by berzosertib + cisplatin in patients with advanced triple-negative breast cancer (TNBC) [72]. Plummer et al. [73] evaluated berzosertib in combination with gemcitabine in patients with advanced NSCLC. The study found that berzosertib plus gemcitabine (ORR 10.5%) did not exhibit significant advantages over gemcitabine monotherapy, but high TMB and LOH scores (30.0%) indicated the notably higher responsiveness of tumors compared to low scores. However, a phase II randomized trial showed that berzosertib added to cisplatin + gemcitabine did not improve the survival benefit of patients with metastatic urothelial carcinoma, with more treatment-related hematologic adverse events [74]. VE-822 inhibited the activation of ATR/CHK1 in esophageal squamous cells, allowing the accumulation of cisplatin-induced DNA damage, which was particularly pronounced in ATM-deficient tumors [75]. In oxaliplatin-resistant rectal cancer mouse models, VE-822 restored the sensitivity of tumor cells to oxaliplatin, and in combination with oxaliplatin, significantly increased the occurrence of single-strand breaks and DSBs, resulting in tumor cell growth suppression [76]. Takeuchi et al. [60] reported that VE-822 reduced the expression of p-CHK1 in HEC-1-B and HEC-6 endometrial cancer cell lines, and combined with DXR, CDDP, or IR, significantly enhanced tumor cell killing, confirming its chemo/radiotherapy sensitization effect. A phase I clinical trial was the first to investigate the use of an ATR inhibitor (M6620) alone or in combination with carboplatin in patients with advanced solid tumors. The results suggested that M6620 monotherapy had a favorable safety profile and brought about complete remission and 29 months of PFS in a patient with metastatic CRC harboring ATM deletion and an ARID1A mutation, but hematologic toxicity was detected when M6620 plus carboplatin at a high dose level was used [77]. A whole-exome sequencing study of primary lesions and paired abdominopelvic metastases in endometrial cancer showed that ARIDIA was frequently mutated in endometrial cancer [78]. The mutation rate of ARIDIA reached 40% in low-grade endometrial cancer [79]. Mutations in the ARIDIA gene directly lead to protein deletion, and compared to other malignancies including colorectal and breast cancers, ARIDIA is most commonly absent in endometrial cancer [80]. This suggests that M6620 monotherapy might exert an antitumor effect in endometrial cancer with ARIDIA mutations, but the specific effects and safety need to be evaluated in clinical trials in selected patients with ARIDIA-mutant endometrial cancer. Thomas et al. [81] evaluated the efficacy and safety of M6620 in combination with topotecan for the treatment of 21 patients with advanced solid tumors, including 1 patient with stage IVB endometrial cancer. The results showed that all patients were generally well tolerated, with two and eight patients achieving partial remission and disease stabilization, respectively, and with endometrial cancer patients maintaining remission for 18 months. Ceralasertib (AZD6738) is an oral ATR inhibitor. Yap et al. [82] reported that ceralasertib yielded a stable disease rate of up to 53% in patients with advanced solid tumors while achieving partial remission in two patients with the loss or downregulation of ATM or SLFN11 protein expression. Kim et al. [83] evaluated the efficacy of ceralasertib plus paclitaxel in refractory cancers, with an overall ORR of 22.7%, and an ORR of 33.3% in 33 patients with anti-PD-1 refractory melanoma. Elimusertib (BAY1895344) is a novel ATR inhibitor that produced powerful efficacy as monotherapy and synergistic antitumor activity when combined with radiotherapy, chemotherapy, or other DDR inhibitors in tumor xenograft models [84]. BAY1895344, in the first-in-human trialm showed good tolerability and powerful inhibitory effects in tumors with DDR deficiency, such as ATM deletion [85]. M4344 is also a potent ATR inhibitor that strengthened the antitumor activity of radiotherapy or chemotherapy in diverse models in vitro and in vivo, and its safety and tolerance need to be further investigated [86]. In addition, Teng et al. [61] found that another ATR inhibitor, ETP-46464, sensitized the radiation treatment in ovarian, cervical, and endometrial cancer cell lines, independent of the P53 status. ATRi and ATMi in combination with radiotherapy resulted in a significantly higher level of apoptosis than ATMi or ATRi plus radiotherapy, which might be related to the activation of ATM induced by radiation, which further activated the ATR-CHK1 pathway. More importantly, ATR inhibitors and platinum generated synergistic cytotoxicity in p53-wild or p53-mutant platinum-resistant tumor cell lines, which provided a great treatment strategy for newly diagnosed or recurrent ovarian, cervical, and endometrial cancers [61]. In brief, ATR inhibitors have preliminary clinical activity and are able to improve the survival benefit of patients with refractory solid tumors to some degree, which may make them competitive candidates for the treatment of recurrent endometrial cancer (regardless of P53 status).

CHK1 is involved in the regulation of S and G2/M checkpoints as well as DNA repair. The inhibition of CHK1 can be an effective antitumor strategy, especially for tumors with defective G1 checkpoints (usually due to P53 loss) [87]. Prexasertib (LY2606368) mainly blocks CHK1 and also inhibits CHK2 to some extent. Prexasertib could effectively decrease the homologous recombinant repair efficiency (more than 55%) of TNBC cells and coordinate with olaparib to augment DNA damage [88]. A phase Ib clinical study demonstrated the acceptable efficacy and safety of prexasertib monotherapy in various squamous cell carcinomas, with an overall clinical benefit rate of 29% at 3 months [89]. However, some studies pointed out some clinical drawbacks of prexasertib. Yang et al. [90] initially explored the combination of prexasertib with radiotherapy + cisplatin or cetuximab for the treatment of patients with locally advanced head and neck squamous cell carcinoma, but the further verification of efficacy was required due to the sample size and follow-up factors. Moore et al. [91] reported during the same period that prexasertib combined with cisplatin, cetuximab, pemetrexed, or 5-fluorouracil provided limited clinical remission and brought about dose-restrictive, reversible hematologic adverse events in patients with advanced or metastatic cancer. Among gynecologic malignancies, prexasertib has been extensively studied in ovarian cancer. An open-label, single-center, proof-of-concept phase II study evaluated its efficacy and safety in patients with BRCA wild-type recurrent high-grade serous ovarian cancer with a PR of 29% [92]. Prexasertib alone produced long-lasting activity in platinum-resistant or refractory relapsed ovarian cancer, regardless of clinical features, BRCA status, or previous treatment status [93], but relatively low clinical efficacy in BRCA wild-type advanced TNBC and extensive-stage small-cell lung cancer (SCLC) [94,95]. In addition, the antitumor activity of prexasertib in combination with PARP inhibitors or PI3K/mTOR inhibitors was preliminarily validated in phase I clinical trials [96,97], and further research is ongoing. Preclinical data indicate that CHK1 inhibitors plus chemotherapy and/or radiation therapy might be a highly effective antitumor strategy. One study reported that the CHK1 inhibitor SAR-020106 intensified radiotherapy-induced DNA damage and apoptosis [98]. In p53-deficient breast cancer cells, the CHK1 inhibitor MK-8776 dramatically improved the cytotoxicity of low-dose doxorubicin therapy with reduced risks of systemic toxicity [99]. In endometrial cancer, Takeuchi et al. [60] calculated the combination index (≤1 to define synergistic effects) using the Talalay–Chou method to evaluate the synergistic effect of an ATR inhibitor (VE-8220) combined with CHK1 inhibitor (AZD7762). The combined indexes in HEC-1-B and HEC-6 endometrial cancer cell lines were 0.43 and 0.28, respectively, suggesting that the co-inhibition of ATR and CHK1 provided excellent antitumor activity in endometrial cancer [60]. In summary, CHK1 inhibitors have shown preliminary antitumor activity in multiple studies, and their combination with other treatments can accelerate tumor cells’ elimination, which also displays some activity in endometrial cancer cell lines, but more evidence is needed to support it due to a lack of relevant clinical studies. 

Wee1 kinase is a key regulator of S and G2/M cell cycle checkpoints and replication stress response [100]. The inhibition of Wee1 kinase abrogated the G2/M cell cycle checkpoint, causing the accumulation of DSBs and programmed cell death [101]. Adavosertib/AZD1775 is the first small-molecule inhibitor of Wee1 kinase [102] and is a relatively mature agent in current research. Takebe et al. [103] determined that the recommended phase II dosage of adavosertib was once daily, and partial remission was observed in 14% of patients with advanced solid tumors (including two patients with endometrial cancer), with CCNE1 overexpression before treatment in two patients. Another multicenter phase II clinical trial investigated adavosertib in refractory solid tumors (including three endometrial carcinomas) characterized by CCNE1 amplification, with an ORR of 27% and improved survival [104]. CCNE1, the gene encoding cyclin E1 protein, has been observed to be overexpressed in many cancers, including endometrial cancer, which results in chromosome instability [105]. CCNE1 is amplified in 8% of endometrioid EC [106], 50% of serous EC [107], and 45% of uterine carcinosarcomas [108]. Cyclin E1 overexpression leads to the premature entry of the cell cycle into the S phase, resulting in increased DSBs and replication stress, which strongly relies on WEE1 to repair damaged DNA in G2/M phase. Therefore, CCNE1 amplification is a biomarker for the application of WEE1 inhibitors in endometrial cancer. Apart from monotherapy, adavosertib in combination with other drugs is also undergoing clinical trials. Keenan et al. [109] added adavosertib to cisplatin in patients with metastatic TNBC who had previously received first-line therapy, with an ORR of 26%. An open-label phase Ib study evaluated the safety and efficacy of adavosertib combined with carboplatin ± paclitaxel in advanced solid tumors in Asia, with 16.7% partial response [110]. However, combination therapy may also increase the occurrence of adverse events. A randomized, double-blind phase II trial reported that adavosertib in combination with paclitaxel + carboplatin improved the ePFS of patients with p53-mutant platinum-sensitive ovarian cancer, but it was accompanied by more frequent treatment-related adverse events [111]. For platinum-resistant/refractory recurrent ovarian cancer, adavosertib plus gemcitabine predominantly extended the PFS, supporting further clinical evaluation [112]. Moreover, adavosertib in addition to chemoradiotherapy had shown initial survival benefits in intermediate/high-risk head and neck squamous cell carcinoma and locally advanced pancreatic cancer, which is in need of validation in a phase II study [113,114]. Until now, only one clinical trial has investigated the application of adavosertib alone in endometrial cancer. Liu et al. [100] assessed the antitumor activity of adavosertib in recurrent serous endometrial carcinoma, with an ORR of 29.4% and a PFS at 6 months of 47.1%, indicating that adavosertib could yield inspiring activity in endometrial cancer. As reviewed above, the Wee1 inhibitor adavosertib is able to ameliorate patients’ survival benefits and has the potential to be a good candidate for the treatment of advanced or recurrent/metastatic endometrial cancer. In addition, p53 mutations in tumors may act as effective predictive markers. 

The content above summarizes the research data of the ATM/CHK2/P53 pathway and ATR/CHK1/WEE1 pathway inhibitors, which are still in the development stage in endometrial cancer, and Table 2 summarizes the research of relevant kinase inhibitors in endometrial cancer, including basic studies and clinical trials. 

### 3.2. Prognostic Biomarker in Endometrial Cancer

There is growing evidence that in addition to being therapeutic targets, DDR pathway gene alternations can be used as prognostic and treatment-response-predictive markers in endometrial cancer. In the following, the prognostic value of several critical proteins involved in the DDR pathway, including p53, ATM, ATR, CHK1, CHK2, and Wee1 in endometrial cancer, is discussed. 

P53 is mutated in most human tumors, with a mutational frequency of more than 90% in serous endometrial carcinoma, which is often associated with a poor prognosis [9]. CNH endometrial cancer is usually accompanied by p53 gene mutations. Subsequently, with the evolution of TransPORTEC [116] and ProMisE [117,118] molecular classifiers, the p53 mutation is commonly used to represent CNH endometrial cancer. Bosse et al. [119] enrolled 381 patients with grade 3 endometrioid carcinoma and divided them into four subgroups according to the molecular typing: POLE-mutation (12.9%), MSI (36.2%), CNL (30.2%), and p53abn (20.7%). The study found that the RFS and OS of p53abn patients were significantly shortened, and the construction of a multivariate cox model confirmed that p53 status was an independent prognostic factor for RFS impairment. Moreover, the PORTEC-3 trial successfully analyzed the influence of molecular classification on adjuvant therapy and disease prognosis in 410 high-risk endometrial cancer cases, and the 5-year RFS of p53abn patients was notably lower than that of the other three subtypes (48% vs. POLE 98%, dMMR 72%, and CNL 74%) [120]. These studies all suggest that p53 gene mutation is closely related to the clinical outcome of endometrial cancer and is a marker of poor prognosis. As mentioned earlier, it is difficult to provide satisfactory antitumor efficacy with immunotherapy alone in patients with p53abn endometrial cancer. Therefore, optimizing adjuvant regimens may facilitate survival benefits in these patients. 

ATM mutations or functional defects may be prognostic markers for endometrial cancer. The prognostic significance of ATM in a variety of tumors had been studied [121,122,123,124]. A TCGA pan-cancer study reported that ATM somatic mutations were most frequent in endometrial cancer [125]. Mhawech-Fauceglia et al. [126] detected the expression of DNA repair protein in 357 cases of endometrial cancer via tissue microarray and found that patients characterized by ATM (+) and p53 (+) or FANCD2 (+) had a remarkably higher risks of recurrence compared with other patients, with a 5-year RFS rate of 80.3%. Furthermore, through comprehensive bioinformatics analysis, Sun et al. [127] discovered that ATM gene mutations could independently predict the OS rate of endometrial cancer, and ATM mutant tumors had a higher level of tumoral neoantigens, which represented more favorable clinical outcomes of patients treated with ICIs [128]. These studies suggest that ATM mutations can be the potential marker for predicting the clinical outcome and response to immune checkpoint blockade in endometrial cancer, but the intrinsic mechanism of ATM mutation and antitumor immune activation should be better described.

Mutations in the ATR gene are most frequent in endometrial cancer compared with other tumors [125]. ATR heterozygous mutations, which may be associated with tumorigenesis and progression, have been widely demonstrated in MSI tumors [129,130,131]. Fang et al. [132] induced genomic instability and chromatin amplification and rearrangement by targeting a single ATR allele in MLH1-deficient HCT116 colon cancer cells and observed the abrogation of CHK1 activation. Moreover, the study found that mice with ATR heterozygous mutations were prone to stillbirth and early-stage tumors. Lewis et al. [133] found that ATR mutations negatively regulated ATR-dependent CHK1 activation and cell cycle arrest after DNA damage, and in ATR-mutated endometrial cancer, ATR-dependent DDR response was inactivated, indicating that ATR mutations play an important role in MSI endometrial cancer. Zighelboim et al. [134] also tried to explore the prognostic significance of ATR in endometrial cancer. A total of 475 eligible patients were divided into an MSI+ group and MSI- group based on their MSI status, and mutations in ATR were assessed via direct sequencing. The results showed that ATR mutations were only observed in endometrial cancers with MSI and were associated with high-grade endometrial cancers, without confirmed correlation with OS or PFS. Therefore, ATR mutations can promote endometrial cancer tumorigenesis in the context of MSI, which may be related to tumor invasiveness, and identifying ATR mutation status is helpful for targeted therapy of endometrial cancer.

According to the TCGA pan-carcinoma analysis, CHK1 mutations were rare in all tumors, and point mutations were relatively more common in endometrial cancer [125]. The high mutation frequency of CHK1 was associated with advanced tumors and might play an important role in tumorigenesis and progression [131]. Dinoi et al. [135] conducted an immunohistochemical analysis on 36 advanced (stage III/IV) serous endometrial cancer samples and found that higher CHK1 expression indicated a decreased risk of disease progression and recurrence. About 6.4% of endometrial cancer cases harbor CHK2 point mutations [125]. Different expression statuses of CHK2 in different tumor types represent diversified prognostic significance. Lee et al. [136] reported that CHK2 deletion indicated advanced tumors and worse disease survival. In addition, through multivariate analysis, CHK2 loss was found to be an independent prognostic factor for adverse outcomes in gastric cancer. In contrast, Eichenauer et al. [137] classified the expression level of CHK2 in prostate cancer into three grades (weak, medium, and strong) through immunohistochemical analysis and found that the high expression of CHK2 was associated with a variety of malignant tumor characteristics and could be used as an independent predictor for the early recurrence of prostate cancer. The different expression levels of CHK2 may have disease-specific prognostic value, but relevant studies have not been carried out in endometrial cancer. In conclusion, the prognostic value of CHK1/2 mutation or abnormal expression in endometrial cancer awaits more data to support it.

The Wee1 gene is scarcely mutated in solid tumors, and about 3.2% of endometrial cancers are characterized by Wee1 point mutations [125]. Multiple research data show that the abnormal expression of Wee1 protein had prognostic value in tumors. In one study, the high expression of Wee1 was related to the poorer survival of patients with malignant melanoma, and knocking out Wee1 caused DNA damage and apoptosis in WM239 (WTp53) and WM45.1 (MTp53) metastatic melanoma cell lines, suggesting Wee1 had a potential prognostic effect independent of p53 status [138]. The high expression of Wee1 could also be observed in vulvar squamous cell carcinoma and was linked to malignant tumor markers (such as lymph node metastasis and poor differentiation) [139]. Slipicevic et al. [140] analyzed the Wee1 protein level in ovarian cancer via immunohistochemistry, which was higher in patients who relapsed after chemotherapy and was associated with reduced OS. The researchers also knocked out Wee1 by siRNA in SKOV3 and OVCAR8 cell lines and found that Wee1 knockout inhibited proliferation in both cell lines. This study suggests that a high level of Wee1 is an independent prognostic marker for ovarian cancer. Furthermore, Ge et al. [141] detected Wee1 expression in CRC samples and found that the expression level of Wee1 mRNA in CRC was significantly upregulated compared with normal tissue and was concerned with tumor metastasis and disappointing prognosis. In TNBC, Wee1 expression was inversely associated with prognosis, but its high expression referred to sensitivity to Wee1 inhibitors [142]. However, the high expression of Wee1 protein indicates better survival outcomes in glioblastoma [143]. In general, the abnormal expression of Wee1, especially high expression, is a marker of poor prognosis in most solid tumors and may also become a predictor for the efficacy of Wee1-targeted therapy. Given that the prognostic significance of Wee1 has not been reported in endometrial cancer, relevant studies need to be conducted in the near future.

The above study found that the alterations in DDR proteins in solid tumors, including endometrial cancer, are closely related to patient survival, which can serve as a marker to predict patients’ prognosis and may guide the targeted therapy with corresponding small-molecule inhibitors. Interestingly, some studies have reported that DDR interacts with antitumor immunity, and corresponding inhibitors may expand the boundary of immunotherapy in endometrial cancer and provide more effective treatment strategies for immune-checkpoint-inhibitor-resistant tumors. Thus, relevant molecular mechanism studies are reviewed below.

## 4. Crosstalk between the DNA Damage Response and Innate and Adaptive Immunity

Increasing data suggest that DDR is related to innate and adaptive immunity in tumors. DDR defects and/or DNA damage increase the tumoral vulnerability to the immune system. Firstly, DDR defects accumulate mutations in the tumor genome, which can act as neoantigens to enhance immunogenicity, thus sensitizing immunotherapy [144]. For example, point mutations caused by dMMR contribute to the production of tumor neoantigens that facilitate tumor immune recognition [145]. Tumors with homologous recombinant repair defects represent higher genomic instability and responsiveness to immunotherapy [146,147]. DDR inhibitors can artificially augment DNA damage and generate DDR defects so that tumor immunogenicity and tumor sensitivity to immunotherapy are increased. Secondly, previous studies have shown that DDR inhibitors trigger tumor immune responses through the cGAS-STING pathway. Severe genomic instability leads to DNA collapse, and fragments of DNA enter the cytoplasm directly or through the formation of micronuclei indirectly, allowing dsDNA to accumulate in the cytoplasm [148]. dsDNA in the cytoplasm is activated by binding with cyclic GMP-AMP synthase (cGAS) and forming a stable cGAS-dsDNA complex, and the activated cGAS catalyzes the synthesis of the second messenger, cyclic avianynylate (cGAMP), which in turn activates the interferon (IFN) gene-stimulating protein (STING). After that, STING migrates to the Golgi apparatus, resulting in the phosphorylation of TANK-binding kinase 1 and IFN regulator 3, further mediating IFN transcription and the expression of multiple inflammatory factors [149]. Subsequent studies have pointed out that the cGAS-STING pathway is defective in some tumors [150], in which DDRi induces the expression of cellular endogenous retroviral elements, activating IFN signaling by triggering dsRNA stress [148]. IFN is a critical bridging factor between innate and adaptive immunity, which is necessary to initiate an immune response in tumors. Type I IFNs (IFN α and IFNβ) activate the JAK1/ STAT pathway, inducing the transcription of the IFN-stimulating factor (ISG), including ISG15, CCL5, and CXCL10 [151]. At the same time, type I IFN is also a stimulatory factor for dendritic cells, which can prolong the retention of tumor antigens in dendritic cells and enhance the expression of major histocompatibility complex class I molecules on the cell surface [152,153,154]. Dendritic cells primarily present exogenous antigens by major histocompatibility complex class I to activate tumor-specific CD8^+^ T cells, which are able to initiate the tumor cell death program [155]. Moreover, type I IFN can promote the recruitment and activation effect of CD8^+^ T cells by stimulating the secretion of chemokines CXCL9 and CXCL10 in dendritic cells [156]. In addition, studies have found that some sorts of DNA damage upregulate the expression of PD-L1 in cancer cells. Additionally, as mentioned earlier, PD-L1 is one of the biomarkers of immunotherapy, which provides a reliable basis for the co-inhibition of DDR and PD1/PD-L1. DSBs, as key exogenous cellular stress molecules, induce higher levels of PD-L1 expression in tumors, and DDR inhibition induces the upregulation of PD-L1 by influencing the DDR pathway to increase the accumulation of DSBs [157]. The expression of PD-L1 requires DNA damage signaling. Additionally, studies have shown that the PD-L1 upregulation requires the activity of ATR/CHK1 kinase in base excision repair-depleted cells [158]. JAKs-STATs-IRF1, a downstream component of the ATR/CHK1 signaling pathway, plays an essential role in the upregulation of PD-L1 mRNA. Activated CD8^+^ T cells in the tumor immune microenvironment secrete IFN-γ, which initiate the JAKs-STATs-IRF1 pathway, further upregulating PD-L1 expression [159]. From this perspective, the ATM/CHK2/WEE1 pathway inhibitors cause the increased expression of PD-L1 in tumor cells and promote immune escape, so they are more suitable for the combined application of PD-1/PD-L1 inhibitors. In conclusion, DDR defects can increase the expression of tumor neoantigens, activate IFN response and enhance CD8^+^ T-cell-mediated specific antitumor immune response, and in some cases upregulate the expression of PD-L1. DDR inhibition may enhance antitumor immunity and improve the efficacy of ICI therapy in cancer, and preclinical data and clinical data of DDR inhibitors combined with ICIs are summarized below. 

## 5. Rationale for “Double-Checkpoint Inhibition” in Endometrial Cancer

DDR defects enhance the activation of tumor immune responses, which provides new ideas for the combinatorial strategy of immunotherapy in endometrial cancer. On the one hand, DDR inhibitors can eliminate tumor cells by inducing DNA damage and apoptosis. On the other hand, DDR inhibitors artificially yield defects in the DDR pathway, which hinder the DNA repair progress, increase the accumulation of damaged DNA, and activate innate immunity and adaptive immunity. Therefore, in addition to intrinsic antitumor activity, DDRi can improve the efficacy of immunotherapy. The new strategy of “double-checkpoint inhibition” may overcome the limitations of ICIs in immune-checkpoint-inhibitor-resistant endometrial cancer, increase the sensitivity to immunotherapy and prevent tumor recurrence or metastasis. The antitumor efficacy of DDR inhibitors has been described in detail above, and the effects on tumor immunity of key ATM/CHK2/P53 pathway and ATR/CHK1/WEE1 pathway inhibitors in DDR, as well as preclinical data of co-inhibition strategies, are reviewed below.

ATM inhibitors induce cell death and are involved in tumor immune responses in different ways. In in vitro and in vivo models of pancreatic cancer, ATM inhibitors increased the expression of type I IFN and then activated immune signaling transduction, while the inhibition of ATM upregulated the expression of PD-L1 and also promoted the response of pancreatic tumors to anti-PD-L1 therapy, indicating the enhanced immunotherapy efficacy by ATM inhibitors and the strong potential of combinational therapy [160]. In ARID1A-deficient tumors with increased CHK2 expression, the inhibition of either ATM or CHK2 could cause cytosolic DNA accumulation by inducing replication stress, followed by the activation of the cGAS-STING signaling pathway that senses cytosolic DNA, leading to an increase in TILs, the activation of the immune response, and enhancement of the antitumor activity of immunotherapy [161]. According to the TCGA database, the co-mutation of ATM and ARID1A genes remarkably expanded the scale of TILs in an endometrial cancer cohort (*n* = 242), and analysis results from another endometrial cancer cohort (*n* = 567) showed that low mRNA levels of ATM and CHK2 in tumors were positively correlated with intensified TILs [161]. An ATM inhibitor, M3541, inhibited the IR-induced initiation of the ATM-dependent DNA repair pathway, inactivated the G1/S cell cycle checkpoint, and induced tumor cell death by disturbing mitosis. In this literature, M3541 plus IR increased chromosomal aberrations and micronucleus formation in A549 cells, activated the cGAS-STING-mediated immune response, and strongly promoted the expression of type I IFN and various inflammatory factors. Furthermore, M3541 enhanced the expression of PD-L1 and increased the sensitivity of A549 cells to NK cell killing by upregulating the expression of NK-cell-activated receptor ligands (ULBP2, CD155, ICAM1, and MICA). Therefore, ATM inhibitors combined with anti-PD-L1 therapy may become a novel treatment method for advanced tumors [162]. Hu et al. [163] found that the hereditary deletion of ATM suppressed tumor growth by the mediation of T cells in mice, and the chemical inhibition of ATM and PD-1 blockade synergistically kill tumor cells. The study also demonstrated an important mechanism that mitochondrial transcription factor A overexpression helped maintain the stability of mitochondrial DNA and avoided the activation of cGAS-STING; hence, ATM inhibitions could cause mitochondrial DNA leakage by downregulating mitochondrial transcription factor A, increasing lymphocyte infiltration in the tumor microenvironment. Shen et al. [164] found that the expression levels of ATM in A549CisR and H157CisR (cisplatin-resistant) cell lines were upregulated, and cisplatin could also induce the upregulation of ATM expression in parental cells. Chemical suppression by CP466722 or ATM knockout by siRNA effectively inhibited epithelial–mesenchymal transformation and tumor metastasis in cisplatin-resistant lung cancer cells and mouse models. However, contrary to previous findings, ATM inhibition downregulated the activity of the JAK/STAT3 pathway and the expression of PD-L1, which requires further investigation of the underlying mechanisms to accelerate the application of its combination with anti-PD-L1 therapy. 

ATR inhibition not only induces tumor growth arrest and apoptosis but also promotes cytosolic DNA formation and immunogenic cell death by replication stress. Combès et al. [76] found that the ATR inhibitor VE-822 plus oxaliplatin prolonged patients’ survival by coordinately enhancing the antitumor immune response of T cells. In ATM-deficient cancer cells, the ATR inhibitor ceralasertib combined with the DNA crosslinker, PBD SG-3199, activated dendritic cells in a STING-IFN-dependent manner, externally fortifying tumor immunogenicity [165]. The activation of the STING pathway can strengthen antitumor immunity. Li et al. [166] identified three STING-related subtypes of SCLC from the RNA-seq dataset: STING-high, STING-median, and STING-low, among which, STING-high tumors were associated with increased immune infiltration and expression of immune checkpoint genes, while the STING-low type had higher activity of DDR and cell-cycle-checkpoint-related pathways. ATM and topology isomerase 1 inhibitors promoted the activation of the type I IFN pathway and the expression of inflammatory factors in STING-low cell lung cancer cell lines. Studies have shown that DNA-damage-induced PD-L1 expression depends on the proficient function of ATR kinase, and although ATR inhibition failed to elevate PD-L1 expression, there are other ways to enhance the antitumor immune response. Sun et al. [167] found that ATR kinase inhibitors attenuated the interaction between PD-1 and PD-L1 by inhibiting PD-L1 expression but sensitized the T-cell killing effect in tumors. Another study showed a significant increase in the expression of cytokine (CCL5, CXCL10, and IFN-β) mRNA and the survival of animals by an ATR inhibitor (BAY1895344) plus anti-PD-L1 therapy, compared with BAY1895344 or anti-PD-L1 therapy alone in prostate cancer mouse models [168].

The inhibition of CHK1/2 can also affect tumor immune response. Chao et al. [169] found that the combination of a CHK1/2 inhibitor (AZD7762) with radiotherapy effectively initiated type I IFN signaling and T-cell response by accelerating immunogenic micronucleus formation and also increased the T-cell infiltration and transduction of immune-activating gene signals, thereby reducing tumor volume. In SCLC models, the CHK1 inhibitor, SRA737, in combination with anti-PD-L1 therapy showed good antitumor activity, which also induced the expression of IFN-β and chemokines (CCL5 and CXCL10), increased the infiltration of CD8^+^ T cells and dendritic cells, and excluded the immunosuppressive cells [170]. Some studies have shown that the expression of PD-L1 in cancer cells depends on CHK1 activity [157], but this does not tell the whole story. In one study, prexasertib significantly enhanced PD-L1 expression in in vitro and in vivo models of SCLC. Moreover, low doses of prexasertib predominantly inhibited tumor growth and produced higher PD-L1 in immune-competent mice compared with immune-deficient mice, indicating that CHK1i induced a stronger immune response in the immune-proficient tumor microenvironment. In addition, although CHKi monotherapy slowed down the tumor growth, it was insufficient to eradicate tumors, and single anti-PD-L1 therapy did not display antitumor activity in the mouse model. Excitingly, prexasertib + anti-PD-L1 treatment significantly regressed tumor lesions in mouse models with a complete response of 6/10. Prexasertib also promoted the infiltration of CD3^+^ T cells, cytotoxic CD8^+^ T cells, and CD44^+^ memory/effector T cells in the lung cancer microenvironment, which was more pronounced after being combined with anti-PD-L1 therapy [171]. Therefore, CHK1 inhibitors can drive the activation of tumor immune responses and can also augment the activity of anti-PD-L1 therapy by upregulating intrinsic PD-L1 expression, which makes CHK1i combined with anti-PD-L1 reasonable for the treatment of endometrial cancer, but more research is required to explore this.

Recent studies have focused on the link between Wee1 inhibitors and tumor immunity to find new choices for combinational therapy strategies. Guo et al. [172] found that the Wee1 inhibitor, AZD1775, increased T-cell infiltration and PD-L1 expression by upregulating the expression of endogenous viral retro-transcriptional elements, stimulating dsRNA stress response and IFN pathway activation. Moreover, the study found that AZD1775 in combination with anti-PD-L1 therapy deterred tumor growth in a CD8^+^ T-cell-dependent manner. In SCLC models, AZD1775 was observed to trigger the activation of the STAT1-IFNγ pathway and increased the expression of PD-L1, and in immunocompetent SCLC mouse models, AZD1775 combined with PD-L1 blockade produced impressive tumor suppression [173]. Wu et al. [174] used the synergistic lethality of Wee1i and ATRi to promote the accumulation of cytosolic dsDNA, induce tumor immune response, and upregulate PD-L1 expression. In addition, PD-L1 blockade in turn enhanced the antitumor activity of Wee1 inhibition combined with ATR inhibition. Similar to other DDR inhibitors, Wee1 inhibitors have been observed to decrease the expression of PD-L1 in some studies. In pancreatic cancer mouse xenograft models, the inhibition of Wee1 or Wee1 + ATM notably hindered tumor proliferation but downregulated the expression of several tumoral immune escape promoters (including PD-L1, CMTM6, CD163 and CXCR2), suggesting a complex link between DDRi and immune response in tumors, which should be elucidated in later research [175]. In all, the combination of Wee1i and immune checkpoint blockade may theoretically provide an effective treatment strategy for recurrent or metastatic endometrial cancer, but relevant research data are lacking, and studies in vitro and in vivo are necessary to evaluate the combinational activity. 

The responsiveness of endometrial cancer to ICIs is considered to be associated with PD-L1 expression, TMB, TILs, etc. DDR inhibition may add DNA damage, resulting in higher TMB and TILs, and upregulate the expression of PD-L1. DDR inhibitors can stimulate innate and adaptive immunity and intensify the efficacy of immunotherapy, thereby avoiding tumor metastasis and drug resistance. Although DDR inhibitors upregulate or downregulate PD-L1 expression in different studies, DDRi plus anti-PD-L1 therapy successfully induces impressive tumor regression. Therefore, the combination of dual checkpoint inhibitors is expected to broaden treatment options for advanced or recurrent/metastatic endometrial cancer.

## 6. The Combination Strategies of DNA Damage Response Pathway Inhibitors and Immune Therapy

Preclinical data support the involvement of DDR inhibitors in tumor immunity and their antitumor potential in combination with immunotherapy. The ultimate goal of the studies is a clinical application to improve the treatment outcome of tumor patients, and the detection of clinical specimens is more supportive of the treatment strategy of double-checkpoint inhibition. Lampert et al. [176] performed multiparametric flow cytometry and RNAseq on paired fresh blood and tissue biopsy specimens of recurrent high-grade ovarian cancer before and after CHK1i treatment, to assess variations in the immune response. Flow cytometry analysis showed that CHK1i enhanced DNA damage and the activation of innate immune responses (mainly increasing monocytes). Human leukocyte DR antigen, a marker of monocytes’ immune activity, and TANK-binding kinase 1, a major marker of STING pathway activation, were both enriched in biopsy tissues and were related to prolonged PFS. RNAseq data analysis also suggested the activation of adaptive immune responses after CHK1i treatment. Several clinical trials reported that DDR inhibitors promoted tumor immunogenicity and immune reactivity, and their combination with immunotherapy is associated with improved clinical benefit. A single-arm, phase II clinical trial showed that in unselected metastatic TNBC, immune-related gene expression and T lymphocytes were highly enriched in tumors of patients who benefited from Wee1 inhibitors in combination with cisplatin [109]. Therefore, clinical data related to immune activation by DDR inhibitors have driven the clinical exploration of DDR inhibitors combined with immunotherapy.

A phase II, single-center, open-label, non-randomized clinical trial evaluated the safety and efficacy of the ATR inhibitor, ceralasertib, in combination with the PD-L1 monoclonal antibody, durvalumab, in patients with advanced gastric cancer. Among 31 patients with advanced gastric cancer who had failed at least one-line chemotherapy, the ORR and disease control rate of combination therapy reached 22.6% and 58.1%, respectively, and the median PFS and OS reached 3 months and 6.7 months, respectively. The study also observed innate immune responses triggered by cytosolic DNA and an increase in lymphocytes such as CD8^+^T cells in responders. However, angiogenesis-related pathways were enriched in the tumor microenvironment of non-responders, and genes involved in DDR, metastasis, and angiogenesis were upregulated in patients with disease progression [177]. Therefore, angiogenesis-related molecular features may contribute to the acquisition of resistance to combinational therapy and tumor progression, which is essential for efficacy analysis in the subsequent clinical trial. Another phase II clinical trial showed that the combination of ceralasertib and durvalumab generated great antitumor activity in advanced or metastatic melanoma that had failed previous anti-PD-1 therapy, with an ORR of 31% and a disease control rate of 63.3%. The median duration of response, median PFS, and median OS were 8.8, 7.1, and 14.2 months, respectively. Additionally, common treatment-related adverse events were of hematologic origin that could be relieved by drug reduction or interruption. Furthermore, tumors with altered DDR pathway genes or immunity-enriched microenvironments have exhibited higher sensitivity to ICIs [178]. The ongoing clinical trial (NCT0368228) is expected to recruit patients with solid tumors, including endometrial cancer, to evaluate the antitumor effects of ceralasertib in combination with durvalumab. In 2021, DO et al. [179] published a clinical study on the application of a CHK1 inhibitor (prexasertib) + PD-L1 monoclonal antibody (LY3300054) in high-grade ovarian cancer and other solid tumors. The study assessed the T-cell subsets and their activation via peripheral blood flow cytometry in patients before and during treatment to analyze the tumor immunity-modulatory effect of prexasertib combined with LY3300054, and observed CD8^+^T-cell activation. In addition, the combination showed tolerance and antitumor activity in patients with specific subtypes of ovarian cancer, which inspired researchers to continuously explore the clinical efficacy of the combinational application of the two inhibitors. 

With the advent of precision medicine era, DDRi has developed rapidly. At present, the combination strategy of DDR inhibitors and ICIs is in its infancy and undergoing investigation. Table 3 summarizes the current ongoing clinical trials of double-checkpoint inhibition, involving NSCLC, melanoma, TNBC, endometrial cancer, ovarian cancer, etc., and the research data are expected to be updated. Considering the development status and limitations of immunotherapy in endometrial cancer, as well as the theoretical basis and clinical evidence of the combined application of DDRi plus ICIs, the combination may overcome immune-checkpoint-inhibitor-resistant tumors and improve the survival and quality of life of patients with advanced or recurrent/metastatic endometrial cancer.

## 7. Summary and Future Perspectives

DDRi combined with ICIs is a new promising strategy for the treatment of advanced or recurrent/metastatic solid tumors, and also has a solid theoretical foundation in endometrial cancer treatment, which will become a hotspot in future research. ICIs have made breakthroughs in the treatment of POLE and partial MSI/dMMR endometrial cancer. However, although the FDA has approved the combination of pembrolizumab and lenvatinib for MSS endometrial cancer, the response of immunotherapy alone in these patients remains unsatisfactory. Therefore, for patients with CNL and CNH tumors, the development of alternative therapeutic modalities is essential to improve immunotherapy outcomes, which may lead to improved patient outcomes compared to the combination of lenvatinib. DDR inhibitors alone or in combination with other agents have shown antitumor activity and are confirmed to modulate tumor immunity in many tumors, so DDRi plus ICIs can be a potentially effective treatment strategy for advanced or recurrent/metastatic endometrial cancer. Therefore, we mainly review the progression, effectiveness and limitations of immunotherapy in endometrial cancer and systematically summarize the research advances of DDRi. Furthermore, the intrinsic relationship between the DDR pathway and innate immunity and adaptive immunity is explored, and the preclinical and clinical data of DDR inhibition related to tumor immunity modulation and immunotherapy sensitization are summarized, which are committed to providing a potent basis for the application of “double-checkpoint inhibition” in immune-checkpoint-inhibitor-resistant endometrial cancer.

However, there are still some limitations to be solved before the clinical application of DDR inhibitors combined with ICIs in endometrial cancer. Firstly, in vivo and in vitro experiments in endometrial cancer models are urgently in need to determine the interaction and related mechanisms of DDRi and tumor immunity. Secondly, effective prognostic and predictive markers should be identified to select the most appropriate patients and optimize the treatment of endometrial cancer. Furthermore, in the future, it will be necessary to investigate the resistance mechanisms involved in combinational therapy, as well as therapeutic drugs that may be effective in reversing tumor resistance, to develop optimum treatment strategies. For example, the activity of the STING pathway is a key factor in the activation of tumor immunity by DDRi, which is generally approved in a large amount of data, and decreased STING activity may contribute to poor response to DDRi therapy. Some studies have found that angiogenesis molecular signatures are enriched in tumors poorly responding to DDRi plus immunotherapy, further causing tumor resistance and progression. The STING agonist Mn^2+^ stimulates dendritic cell and macrophage activation, promotes tumor antigen presentation, and enhances NK cell and CD8^+^ T-cell activation. In mouse models, Mn^2+^ plus anti-PD-1 therapy synergistically inhibits tumor growth with a lower dose of ICIs [180]. For patients with endometrial cancer treated with DDRi + ICI, the addition of Mn^2+^ may reduce the intake of drugs without increasing extra toxicity, while generating stronger antitumor activity. Last but not the least, we should pay more attention to the adverse effects of dual inhibitors and try to maximize the antitumor effect with the lowest drug dose, so as to avoid the adverse effects of drugs that may lead to the failure of an experiment.

In conclusion, DDR inhibitors can enhance the efficacy of immunotherapy and overcome the therapeutic limitations of immunotherapy in insensitive endometrial cancer. However, we should first clarify the evidence and molecular mechanism of DDR inhibition in the regulation of immune response in endometrial cancer, especially immune-checkpoint-inhibitor-resistant diseases, to provide feasible therapeutic targets for subsequent clinical research. Secondly, screening effective predictive markers of treatment response can help select sensitive patients and lay a reliable basis for the application of DDR inhibitors in combination with ICIs in endometrial cancer to achieve precise and individualized treatment. Finally, more clinical trials should be carried out in endometrial cancer for the DDR pathway and its specific small-molecule targeted drugs, further optimizing the efficacy of combinational therapy.

## Figures and Tables

**Table 1:** Clinical trials of immune checkpoint inhibitors in endometrial cancer.

Trial	ICIs	Research Object/Sample Size	Efficacy Index	Phase	Status	References
NCT02054806 (KEYNOTE-028)	Pembrolizumab	Advanced PD-L1-positive endometrial cancer	ORR 13%,	1b	Published	[28]
	(Keytruda)		PR 13%,			
		(*n* = 24.23 evaluable)	SD 13%,			
			Median PFS 1.8 months,			
			PFS—6 months 19.0%,			
			PFS—12 months 14.3%,			
			OS—6-month rate 67.0%,			
			OS—12-month rate 51.0%			
NCT02628067	Pembrolizumab	Advanced MSI-H/dMMR tumors (including endometrial cancer)	ORR 48%,	2	Published	[29]
(KEYNOTE-158)	(Keytruda)		CR 14%,			
			PR 34%,			
		(*n* = 90.79 evaluable)	SD 18%,			
			Median PFS 13.1 months			
NCT02899793	Pembrolizumab	Recurrent MSI-H endometrial cancer with Lynch-like vs. MLH-1 methylated characteristics	Overall ORR 58%	2	Published	[30]
	(Keytruda)		Lynch-like:			
			PFS—3-year rate 100%,			
			OS-3-year rate 100%			
		(*n* = 25)	MLH-1 methylated:			
			PFS—3-year rate 30%,			
			OS—3-year rate 43%			
NCT02715284	Dostarlimab	Recurrent or advanced dMMR endometrial cancer	ORR 42.3%,	1	Published	[14]
(GARNET)	(TSR-042)		CR 12.7%,			
		(*n* = 104.71 evaluable)	PR 29.6%,			
			Median PFS 8.1 months,			
NCT02465060	Nivolumab	dMMR non-colorectal cancers (including 13 endometrioid endometrial adenocarcinomas and 4 uterine carcinosarcomas)	ORR 36%,	2	Published	[17]
(NCI-MATCH/EAY131)	(Opdivo)		SD 21%,			
			PFS—6 months 51.3%,			
			PFS—12 months 46.2%,			
		(*n* = 42)	PFS—18 months 31.4%,			
			Median OS 17.3 months			
NCT02982486	Nivolumab	Non-resectable sarcoma and endometrial carcinoma	PR, CR,	2	Unknown	-
	(Opdivo),		Median PFS, PFS—12 weeks, PFS—24 weeks, OS—3 years			
	Ipilimumab	(*n* = 60, estimated enrollment)				
	(Yervoy, CTLA-4 inhibitor)					
NCT04106414	Nivolumab	Endometrial adenocarcinoma and endometrial carcinosarcoma	ORR	2	Active, not recruiting	-
	(Opdivo),					
	BMS–986205	(*n* = 24, actual enrollment)				
	(IDO-1 inhibitor)					
NCT02791334	LY3300054	Advanced refractory MSI-H/dMMR solid tumors (including 14 endometrial cancers)	LY3300054 (*n* = 40):	1b	Published	[18]
	(PD-L1 inhibitor)		ORR 32.5%			
	LY3321367		CR 12.5,			
	(TIM-3 inhibitor)		PR 20%,			
		(*n* = 82)	SD 27.5%,			
			PFS rate 62.5%,			
			Median PFS 7.4 months			
			LY3300054 + LY3321367 (*n* = 42):			
			ORR 45%,			
			CR 10%,			
			PR 35%,			
			SD 25%,			
			PFS rate 55%,			
			Median PFS 7.6 months			
NCT03015129	Durvalumab (Imfinzi)	Advanced dMMR and pMMR endometrial cancer	dMMR (*n* = 36):	2	Published	[26]
			ORR 47%			
		(*n* = 71)	PR 30.6%,			
			PFS—6 months 53%,			
			Median PFS 8.3 months,			
			OS—12-month rate 71%			
			pMMR (*n* = 35):			
			ORR 3%,			
			PR 3%,			
			PFS—6 months 14%,			
			Median PFS 1.8 months,			
			OS-12-month rate 51%			
NCT02912572	Avelumab	Recurrent/persistent dMMR and pMMR endometrial cancer	31 included in analysis:	2	Published	[31]
	(BAVENCIO)		dMMR (*n* = 15):			
		(*n* = 33)	ORR 26.7%,			
			CR 6.7%,			
			PR 20%,			
			PFS—6-month rate 40%,			
			Median PFS 4.4 months			
			pMMR (*n* = 16):			
			ORR 6.25%,			
			CR 0%,			
			PR 6.25%,			
			PFS—6-month rate 6.25%,			
			Median PFS 1.9 months,			
			Median OS 6.6 months			

CR, complete response; CTLA-4, cytotoxic T lymphocyte-associated antigen-4; dMMR, mismatch repair deficient; ICI, immune checkpoint inhibitor; IDO-1, indoleamine 2,3-dioxygenase 1; MSI-H, microsatellite instability—high; ORR, objective response rate; OS, overall survival; PFS, progression-free survival; pMMR, mismatch repair proficient; PR, partial response; SD, stable disease.

**Table 2:** Preclinical and clinical data of DNA damage response inhibitors in endometrial cancer.

DDR Target	DDR Inhibitor	Research Object/Sample	Research Type	Phase	Efficacy Index	Status	References
ATM	KU60019	Endometrial cancer cells (HEC-1-B, HEC-6)	Preclinical	-	IC50 20 µM	Published	[60]
ATM	KU55933	Endometrial cancer cells (HEC-1-B, KLE)	Preclinical	-	LD50 38.3 ± 7.6 µM	Published	[61]
ATR	ETP-46464	Endometrial cancer cells (HEC-1-B, KLE)	Preclinical	-	LD50 10.0 ± 8.7 µM	Published	[61]
ATR	VE822	Endometrial cancer cells (HEC-1-B, HEC-6)	Preclinical	-	IC50 1.5 µM	Published	[60]
ATR	Berzosertib (M6620/VX-970)	Advanced solid tumors (including 1 endometrial cancer)	Clinical	1	PR continuing at 18 months	Published	[81]
		(*n* = 1)					
CHK1	GDC-0575	Refractory solid tumors (including 4 endometrial cancers)	Clinical	1	SD or PR 66%	Published	[115]
		(*n* = 102, 90 evaluable)					
ATR	VE822	Endometrial cancer cells (HEC-1-B, HEC-6)	Preclinical	-	CI in HEC-1-B: 0.43	Published	[60]
CHK1	AZD7762				CI in HEC-6: 0.28		
Wee1	Adavosertib (AZD1775)	Advanced solid tumors (including 3 endometrial cancers and 1 uterine carcinosarcoma)	Clinical	1	PR 14.3%,	Published	[103]
		(*n* = 42)			median DOR 4.9 months,		
					SD 47.6%		
Wee1	Adavosertib (AZD1775)	Advanced or refractory malignant solid neoplasm (including 3 uterine tumors)	Clinical	2	ORR 27%,	Published	[104]
		(*n* = 30)			median PFS 4.1 months,		
					median OS 9.9 months		
Wee1	Adavosertib (AZD1775)	Recurrent uterine serous carcinoma	Clinical	2	ORR 29.4%,	Published	[100]
		(*n* = 35, 34 evaluable)			PFS6 47.1%,		
					median PFS 6.1 months		

ATM, ataxia telangiectasia mutated; ATR, ataxia telangiectasia and Rad3-related; CHK1, checkpoint kinase 1; CI, combination index; DDR, DNA damage response; DOR, duration of response; IC50, the half maximal inhibitory concentration; LD50: lethal dose resulting in 50% mortality; ORR, objective response rate; OS, overall survival; PFS, progression-free survival; PR, partial response; SD, stable disease.

**Table 3:** Ongoing clinical trials pertaining to DNA damage response inhibitors in combination with immune checkpoint inhibitors.

Targets	Inhibitors	Research Object/Sample Size	Phase	Status	NCT Number
ATR,	Ceralasertib,	Advanced or metastatic non-small-cell lung cancer	3	Recruiting	NCT05450692
PD-L1	Durvalumab				
		(*n* = 580)			
ATR,	Ceralasertib,	Melanoma	2	Recruiting	NCT05061134
PD-L1	Durvalumab	(*n* = 195)			
ATR,	Ceralasertib,	Triple-negative breast cancer	2	Recruiting	NCT05582538
PD-L1	Durvalumab	(*n* = 37)			
ATR,	Ceralasertib,	Solid tumors (including endometrial cancer)	2	Recruiting	NCT03682289
PD-L1	Durvalumab				
		(*n* = 89)			
ATR,	Ceralasertib,	Advanced solid malignancies-HNSCC, ATM Pro/Def NSCLC, gastric, breast and ovarian cancer	1/2	Recruiting	NCT02264678
PD-L1	Durvalumab				
		(*n* = 330)			
ATR,	Ceralasertib,	Non-small-cell lung cancer	2	Recruiting	NCT03334617
PD-L1	Durvalumab	(*n* = 570)			
ATR,	Ceralasertib,	Non-small-cell lung cancer	2	Recruiting	NCT03833440
PD-L1	Durvalumab	(*n* = 120)			
ATR,	Ceralasertib,	Advanced solid tumors	1	Recruiting	NCT05514132
PD-L1	Durvalumab	(*n* = 12)			
ATR,	Elimusertib,	Advanced or recurrent head and neck cancer	1	Recruiting	NCT04576091
PD-1	Pembrolizumab				
		(*n* = 37)			
ATR,	M1774,	Metastatic or locally advanced unresectable solid tumors	1	Recruiting	NCT05396833
IC	Unknown	(*n* = 72)			

ATM, ataxia telangiectasia mutation; ATR, ataxia telangiectasia and Rad3-related; HNSCC, head and neck squamous cell carcinoma; IC, immune checkpoint; NSCLC, non-small-cell lung cancer; PD-1, programmed cell death protein 1; PD-L1, programmed cell death ligand 1.

## Data Availability

Not applicable.
